# Approaches for difficult-to-induce-seizures electroconvulsive therapy cases (DEC): a Japanese expert consensus

**DOI:** 10.1186/s12991-024-00543-9

**Published:** 2025-01-12

**Authors:** Yoshiteru Takekita, Taro Suwa, Kazuyuki Yasuda, Hirotsugu Kawashima, Wataru Omori, Naoki Kurimoto, Takashi Tsuboi, Takamasa Noda, Nobuatsu Aoki, Ken Wada, Ken Inada, Minoru Takebayash, Expert Consensus Development Working Group, Electroconvulsive Therapy Committee, Japanese Society of General Hospital Psychiatry

**Affiliations:** 1 Department of Neuropsychiatry, Faculty of Medicine, Kansai Medical University, 10-15 Fumizono-Cho, Moriguchi, Osaka 570-8506 Japan; 2 Department of Psychiatry, Kyoto University Hospital, 54 Shogoin-Kawaharacho Sakyo-Ku, Kyoto, 606-8397 Japan; 3 Faculty of Medicine, Department of Neuropsychiatry, University of Yamanashi, 1110 Shimokato, Chuo, Yamanashi 409-3898 Japan; 4 Department of Psychiatry and Neurosciences, Hiroshima University, Kasumi, Minami‑ku, Hiroshima, 734‑8551 Japan; 5 Shigasato Hospital, 1-18-41 Shigasato, Otsu, Shiga 520-0006 Japan; 6 Department of Neuropsychiatry, Kyorin University School of Medicine, 6-20-2 Shinkawa, Mitaka-Shi, Tokyo, 181-8611 Japan; 7 Department of Psychiatry, National Center of Neurology and Psychiatry, 4-1-1, Ogawahigashi Kodaira, Tokyo, 187-8551 Japan; 8 Psychiatry and Mental Health, University of New South Wales, Hospital Road, Randwick, NSW 2031 Australia; 9 Black Dog Institute, Hospital Road, Randwick, NSW 2031 Australia; 10 Department of Psychiatry, Hiroshima Citizens Hospital, Hiroshima City Hospital Organization, 7-33 Moto-Machi, Naka-Ku, Hiroshima, Japan; 11 Department of Psychiatry, Kitasato University School of Medicine, 1-15-1 Kitasato, Minami-Ku, Sagamihara, Kanagawa 252-0374 Japan; 12 Department of Neuropsychiatry, Faculty of Life Sciences, Kumamoto University, 1-1-1 Honjo, Chuo-Ku, Kumamoto, 860-8556 Japan

**Keywords:** Electroconvulsive therapy, Seizure threshold, Difficult-to-induce-seizures electroconvulsive therapy cases, Expert consensus

## Abstract

**Background:**

Seizure threshold increases with age and the frequency of electroconvulsive therapy (ECT). Therefore, therapeutic seizures can be difficult to induce, even at maximum stimulus charge with available ECT devices. Such cases are known as difficult-to-induce-seizures electroconvulsive therapy cases (DECs). However, no clinical guidelines exist for DECs; thus, clinicians often face difficulties determining treatment strategies. This study aimed to obtain a consensus among clinical experts regarding the treatment of DECs.

**Methods:**

We asked Japanese ECT experts to rate 14 approaches under six conditions of DECs on a 9-point Likert scale (1 = “disagree” to 9 = “agree”). Based on responses from 195 experts, the approaches were classified as first-line (95% confidence interval mean ≥ 6.5), second-line (mean, 3.5–6.5), or third-line strategies (mean < 3.5). Approaches rated 9 points by at least 50% of the respondents were considered “treatments of choice.”

**Results:**

To avoid difficult seizure induction, dose reduction of benzodiazepine receptor agonist (BZRA) (8.33 ± 1.25), dose reduction or discontinuation of antiepileptic drugs (AEDs) or other drugs that may make seizure induction difficult (8.16 ± 1.18), and ensure hyperventilation (7.95 ± 1.47) were classified as treatments of choice. First-line treatment strategies were BRZA discontinuation (7.89 ± 1.45), stimulation timing adjustment (7.00 ± 2.00), and anesthetic dose reduction (6.93 ± 1.94). Dose reduction or discontinuation of AEDs or other drugs that might make seizure induction difficult and ensure hyperventilation were the treatments of choice across all patient conditions. The results of rating approaches for patients with mood disorders and schizophrenia were similar, with differences observed among the approaches for patients with catatonia, high risk of cognitive impairment, and cardiovascular events.

**Conclusions:**

ECT expert recommendations are useful and can assist in clinical decision-making. Our results suggest that while some strategies are applicable across all conditions, others should be tailored to meet the specific needs of patients. These recommendations should be further evaluated in future clinical studies.

**Supplementary Information:**

The online version contains supplementary material available at 10.1186/s12991-024-00543-9.

## Background

Electroconvulsive therapy (ECT) is a neuromodulation technique that has been widely used for treating psychiatric disorders, including depression, bipolar disorder, catatonia, and schizophrenia, for the longest period. Typically, 6–12 ECT sessions are administered during the acute phase of each condition. However, if ECT is the only method for maintaining remission or a symptomatically stable condition, continuous or maintenance ECT may be administered [1].

Although there is considerable individual variation and differences among reports, the seizure threshold may vary with factors such as age, electrode placement, and the course of treatment [2–6], leading to cases referred to as difficult-to-induce-seizures electroconvulsive therapy cases (DECs). DECs are patients in whom therapeutic seizures are difficult to induce, even when the maximum stimulus charge is delivered with available ECT devices [7]. The DECs encompasses situations where seizures are not induced, as well as a deterioration in seizure quality, such as reduced duration and amplitude of the spike-and-wave phase and poorer post-ictal suppression [8]. This presents a major challenge in clinical practice since the effects of ECT may originate from the induction of generalized seizures during the early stages of treatment [9]. Poor seizure quality, as seen in electroencephalography, is associated with suboptimal clinical outcomes in depression [10]. Furthermore, cerebral blood flow differs between patients with well-generalized and those with inadequate seizures [11]. When the effect of ECT decreases, issues such as prolonged hospitalization, delayed remission, and worsening comorbidities may arise, along with an increased seizure threshold due to additional ECT sessions. Globally, techniques to lower seizure thresholds during ECT and prolong seizure duration have been extensively investigated [8]. Innovations include the use of concomitant drugs or premedications for ECT, anesthesia-related techniques, such as the use of intravenous anesthetics, and stimulation techniques associated with electrode placement and pulse width. In Japan, Thymatron® System IV (Somatics, Lake Bluff, IL) with a maximum stimulus charge of 504 mC had been the only device approved for ECT until December 2023 when a device with a maximum stimulus charge of 1008 mC was approved. In addition, bilateral (BL) electrode placement, which is associated with a higher seizure threshold than right unilateral (RUL) electrode placement [12], is used in 75% of cases [13]. Therefore, many clinicians administering ECT in Japan encounter DECs and explore approaches to deal with them [14].

Despite various approaches for DECs, the absence of treatment guidelines, limited large-scale clinical studies based on robust designs, and the lack of direct comparisons between approaches make treatment decisions challenging. The opinions of frontline experts, based on clinical experience, offer practical insights into these issues, which have not been adequately addressed in previous studies. Thus, this study aimed to reach a consensus among Japanese ECT experts on the best approach that should be used in managing DECs in various clinical scenarios.

## Material and methods

### Study design

A working group of 11 ECT experts (all qualified psychiatrists with at least 10 years of experience in ECT and authors of published ECT-related articles in international journals) was established by the Electroconvulsive Therapy Committee of the Japanese Society of General Hospital Psychiatry (the only committee in Japan that grants permissions for ECT training institutions and organizes ECT seminars). The group identified six clinical questions regarding approaches used when seizure induction is difficult during ECT. The listed approaches were based on evidence from the literature and clinical practices in Japan.

The survey period was from March 1 to April 15, 2023, and involved psychiatrists who had participated in ECT training seminars sponsored by the Japanese Society of Psychiatry and Neurology, the Japanese Society of General Hospital Psychiatry, the Japanese Association for Emergency Psychiatry, and the Japan Psychiatric Hospitals Association. The survey also included those affiliated with any of these facilities: facilities designated for ECT training by the Japanese Society of General Hospital Psychiatry, facilities with members of the Electroconvulsive Therapy Committee of the Japanese Society of General Hospital Psychiatry, and facilities that administer ECT to at least 10 patients or perform ECT at least 100 times annually. Experts who met these criteria were invited to participate in an email-based questionnaire survey.

Participants were presented with premises specifying the use of the most standard ECT device and treatment techniques in Japan, as follows: “*This questionnaire is designed based on the scenario that ECT is typically administered with bilateral electrode placement under propofol anesthesia at a pulse width of 0.5 ms and the half-age dosing strategy, using the Thymatron® device (maximum stimulus charge: 504 mC), a pulse-wave therapeutic device approved in Japan. Please, imagine a situation where all the approaches presented in the questions are applicable, and answer the following questions by selecting a number between 1 and 9.*” The experts rated each selected approach on a 9-point Likert scale (1 = strongly not recommended to 9 = strongly recommended). Additional Files 1 and 2 show the clinical questions, six clinical conditions in which DECs were placed, and 14 approaches. The survey was completed in approximately 15 min, and participation was voluntary, without any incentives. Informed consent was obtained via the questionnaire from the experts regarding their participation in the research, provision of research-related information, protection of personal information, management of information, and disclosure of information regarding the research.

In addition, participants were asked to provide information on their years of medical practice, clinical experience with ECT, and affiliated medical institutions.

This study was approved by the Institutional Review Board for Clinical Research of Kansai Medical University Medical Center (2,022,275).

### Statistical analysis

For each approach, the mean value, standard deviation, 95% confidence interval (CI), and number of respondents were calculated according to rating categories (not recommended: 1–3 points, neutral: 4–6 points, recommended: 7–9 points). Regarding each selected approach, similar to the method employed by Sakurai et al. [15], Pearson's chi-squared test was used to compare the number of respondents between the three rating categories. A p-value ≥ 0.05 indicated that "no consensus" was reached on the corresponding clinical question, and the selected approach was controversial.

Expert consensus was interpreted using the method reported by Allen et al. [16]. Approaches with a 95% CI ≥ 6.5 were regarded as "first-line strategies," indicating that expert consensus was reached under certain conditions. Approaches rated 9 points by at least 50% of the respondents were classified as "treatments of choice” (expressed as “Best” in the table), signifying particularly strong recommendations as first-line strategies. Approaches with a 95% CI ≥ 3.5 were defined as "second-line strategies," suitable for patients who do not respond to first-line strategies. Approaches with a 95% CI < 3.5 were defined as "third-line strategies," which are generally inappropriate or used only when other approaches fail.

## Results

### Characteristics of the participants

Responses were obtained from 195 ECT experts from 117 medical institutions in Japan. These included 51 university hospitals (43.6%), 25 general hospitals (21.4%), and 41 psychiatric hospitals (35.0%). None of the participating physicians were affiliated with regional clinics or other facilities. Of the experts who responded to the questionnaire, 29 were excluded from the analysis because they did not meet the criteria for facilities or had not participated in training seminars. Among the 166 valid respondents, the mean durations of medical practice and clinical experience with ECT were 17.8 ± 8.4 and 13.3 ± 8.5 years, respectively.

### Approaches that should be constantly performed to avoid situations where seizure induction is difficult (Fig. 1)

**Fig. 1:**
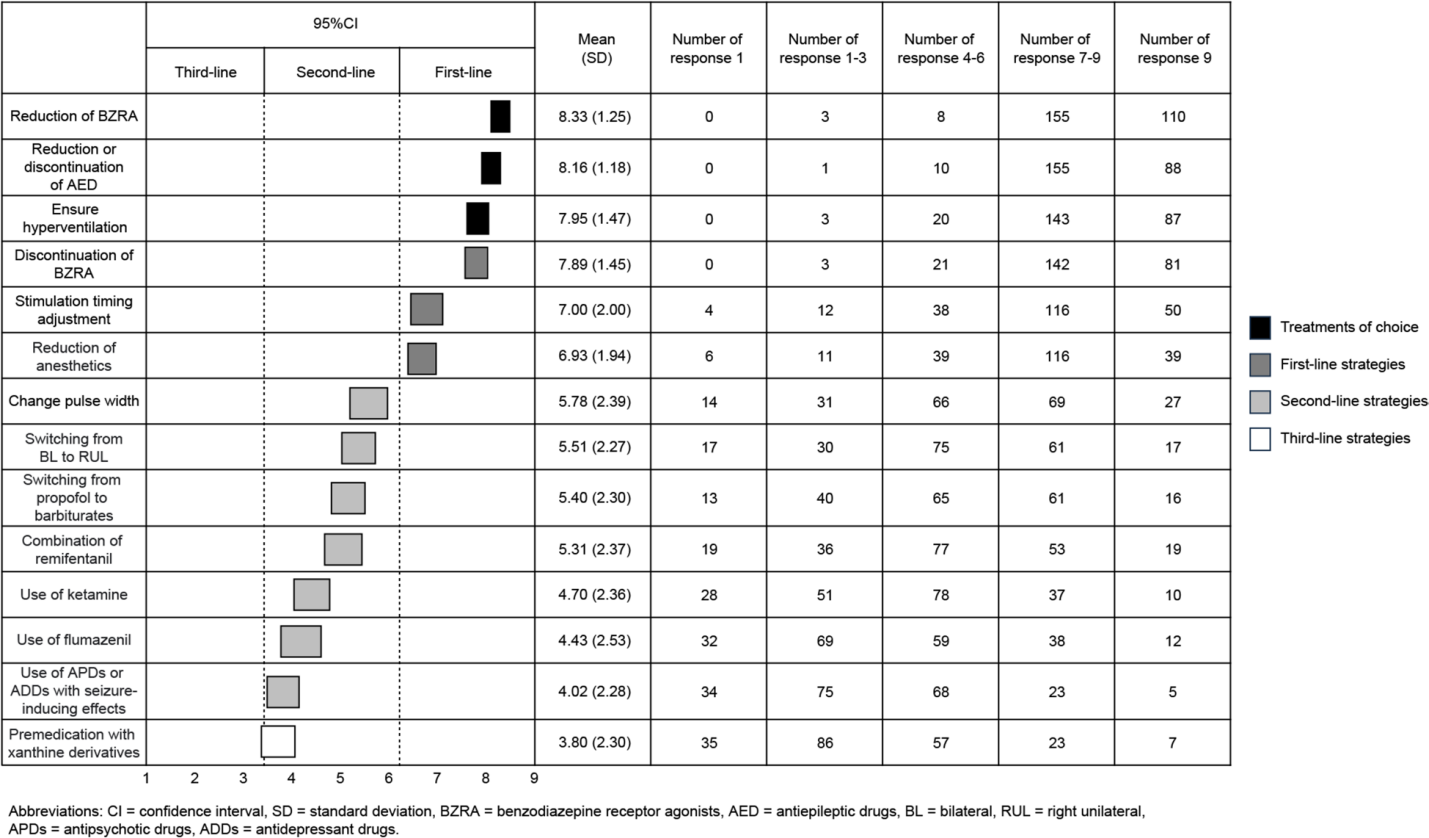
Figure 1. Q1) Approaches that should be constantly performed to avoid situations that make seizure induction difficult

The following approaches were classified as treatments of choice to prevent difficulties in seizure induction: dose reduction of benzodiazepine receptor agonist (BZRA: 8.23 ± 1.25; rated 9 points by 66.3%), dose reduction or discontinuation of antiepileptic drugs (AEDs) or other drugs that may make seizure induction difficult (8.16 ± 1.18; rated 9 points by 53.0%), and ensuring hyperventilation (7.95 ± 1.47; rated 9 points by 52.4%). The approaches classified as first-line strategies were discontinuation of BZRA (7.89 ± 1.45), stimulation timing adjustment (7.00 ± 2.00), and dose reduction of anesthetics (6.93 ± 1.94). Premedication with xanthine derivatives (3.80 ± 2.30) was classified as a third-line strategy. All other approaches were classified as second-line strategies.

### ***Approaches for DECs with mood disorders (***Additional File 3***)***

For DECs with mood disorders, the following approaches were classified as treatments of choice: BZRA dose reduction (8.35 ± 1.16; rated 9 points by 63.9%), ensuring hyperventilation (8.14 ± 1.38; rated 9 points by 62.0%), and AED dose reduction or discontinuation (8.12 ± 1.17; rated 9 points by 53.6%). The approaches classified as first-line strategies were BZRA discontinuation (7.98 ± 1.33), stimulation timing adjustment (7.23 ± 1.91), and anesthetic dose reduction (7.24 ± 1.84). No consensus was reached on the use of flumazenil (4.86 ± 2.43). All other approaches were classified as second-line strategies.

### ***Approaches for DECs with schizophrenia (***Additional File 4***)***

For patients with schizophrenia receiving DEC, the following approaches were classified as treatments of choice: BZRA dose reduction (8.36 ± 1.19; rated 9 points by 65.1%), ensuring hyperventilation (8.15 ± 1.36; rated 9 points by 62.0%), and AED dose reduction or discontinuation (8.14 ± 1.19; rated 9 points by 54.2%). The approaches classified as first-line strategies were BZRA discontinuation (7.98 ± 1.40), anesthetic dose reduction (7.37 ± 1.70), and stimulation timing adjustment (7.23 ± 1.96). No consensus was reached on the use of flumazenil (4.89 ± 2.47) and psychotropic drugs (e.g., antipsychotic drugs [APDs] or antidepressant drugs [ADDs]) with seizure-inducing potential (4.85 ± 2.45). All other approaches were classified as second-line strategies.

### ***Approaches for DECs with catatonia (***Additional File 5***)***

For these patients, the following approaches were classified as treatments of choice: dose reduction or discontinuation of AEDs (8.15 ± 1.22; rated 9 points by 54.8%) and ensuring hyperventilation (8.12 ± 1.42; rated 9 points by 61.4%). The approaches classified as first-line strategies were stimulation timing adjustment (7.30 ± 1.97), anesthetic dose reduction (7.28 ± 1.83), and BZRA dose reduction (6.92 ± 1.93). No consensus was reached on the use of flumazenil (5.40 ± 2.67). All other approaches were classified as second-line strategies.

### ***Approaches for DECs at high risk of cognitive impairment (***Additional File 6***)***

For DECs with a high risk of cognitive impairment, the following approaches were classified as treatments of choice: BZRA dose reduction (8.46 ± 1.01; rated 9 points by 67.5%), BZRA discontinuation (8.35 ± 1.12; rated 9 points by 63.3%), AED dose reduction or discontinuation (8.13 ± 1.20; rated 9 points by 52.4%), and ensuring hyperventilation (8.09 ± 1.49; rated 9 points by 62.0%). The approaches classified as first-line strategies were anesthetic dose reduction (7.33 ± 1.77), stimulation timing adjustment (7.28 ± 1.90), and switching from BL electrode placement to RUL electrode placement (7.20 ± 2.26). No consensus was reached on the use of flumazenil (4.72 ± 2.55). All other approaches were classified as second-line strategies.

### ***Approaches for DECs at high risk of cardiovascular events (***Additional File 7***)***

For DECs with a high risk of cardiovascular events, the following approaches were classified as treatments of choice: BZRA dose reduction (8.34 ± 1.14; rated 9 points by 61.4%), AED dose reduction or discontinuation (8.19 ± 1.20; rated 9 points by 53.0%), and ensuring hyperventilation (7.91 ± 1.66; rated 9 points by 55.4%). The approaches classified as first-line strategies were BZRA discontinuation (8.01 ± 1.31), stimulation timing adjustment (7.25 ± 1.90), and anesthetic dose reduction (7.04 ± 1.98). The use of psychotropic drugs (e.g., APDs or ADDs) with potential seizure-inducing effects (3.70 ± 2.21) and premedication with xanthine derivatives (3.32 ± 2.19) were classified as third-line strategies. All other approaches were classified as second-line strategies.

### ***Comparison between patient groups (***Table 1***)***

**Table 1:** Summary of the consensus on the use of the approaches in difficult-to-induce-seizures electroconvulsive therapy cases according to the conditions

	Procedures to be performed constantly	Mood disorder	Schizophrenia	Catatonia	High risk of cognitive dysfunction	High of cardiovascular events
Discontinuation of BZRA	1st	1st	1st	2nd	Best	1st
Reduction of BZRA	Best	Best	Best	1st	Best	Best
Use of flumazenil	2nd	no consensus	no consensus	no consensus	no consensus	2nd
Premedication with xanthine derivatives	3rd	2nd	2nd	2nd	2nd	3rd
Reduction or discontinuation of AED	Best	Best	Best	Best	Best	Best
Use of APDs or ADDs with seizure-inducing effects	2nd	2nd	no consensus	2nd	2nd	3rd
Reduction of anesthetics	1st	1st	1st	1st	1st	1st
Stimulation timing adjustment	1st	1st	1st	1st	1st	1st
Switching from propofol to barbiturates	2nd	2nd	2nd	2nd	2nd	2nd
Combination of remifentanil	2nd	2nd	2nd	2nd	2nd	2nd
Use of ketamine	2nd	2nd	2nd	2nd	2nd	2nd
Ensure hyperventilation	Best	Best	Best	Best	Best	Best
Change pulse width	2nd	2nd	2nd	2nd	2nd	2nd
Switching from BL to RUL	2nd	2nd	2nd	2nd	1st	2nd

BZRA benzodiazepine receptor agonists, AED antiepileptic drugs, BL bilateral, RUL right unilateral, APDs antipsychotic drugs, ADDs antidepressant drugs

The only treatments of choice in common across all patient conditions were reduction or discontinuation of AEDs and ensuring hyperventilation. BZRA reduction was classified as the treatment of choice in all patient groups except patients with catatonia. Discontinuation of BZRA was classified as the treatment of choice only in patients at high risk of cognitive impairment, whereas it was classified as a second-line strategy in patients with catatonia. Anesthetic reduction and stimulation timing adjustments were classified as first-line strategies for all groups. Switching from BL to RUL was classified as a first-line strategy in patients at a high risk of cognitive impairment, whereas it was classified as a second-line strategy in other groups. Other approaches were largely classified as second-line strategies, although the use of psychotropic drugs that could induce seizures, pretreatment with xanthine derivatives, and the use of flumazenil were classified as third-line strategies or lacked consensus.

## Discussion

To our knowledge, this is the first study to report expert ratings on practical approaches for DECs in clinical settings. The key approaches consistently recommended to prevent difficult seizure induction were dose reduction or discontinuation of AEDs and ensuring hyperventilation, classified as treatments of choice. First-line strategies included dose reduction of anesthetics and stimulation timing adjustment, whereas second-line strategies were switching anesthetics from propofol to barbiturates, the combination use of remifentanil, switching anesthetics to ketamine (alone or in combination), and adjusting pulse width (Table 1). Other approaches were rated differently depending on patient conditions and underlying diseases.

Generally, when clinical guidelines are developed, recommendations for treatment and approaches are often evaluated and described based on evidence. Among the 14 approaches investigated in this survey, meta-analyses with the highest level of evidence have shown that approaches associated with improved outcomes, such as reduced seizure threshold and prolonged seizure duration, include the combined use of remifentanil [17], switching anesthetics to ketamine [18], BL electrode placement to RUL electrode placement [19, 20], and stimulation timing adjustment [21]. In addition, interventions that have been demonstrated by randomized controlled trials (RCTs) to be effective for improving outcomes associated with seizure induction include premedication with xanthine derivatives (specifically caffeine) [22–24], anesthetic dose reduction [25, 26], and pulse width adjustment [27, 28]. In contrast, meta-analyses and RCTs have not shown significant advantages for switching anesthetics from propofol to barbiturates [29, 30] (evaluated by meta-analyses) or ensuring hyperventilation [31–34] (evaluated by RCTs). Furthermore, theoretical approaches supported only by case reports include BZRA discontinuation [35, 36], BZRA dose reduction [3, 35–37], flumazenil use [38, 39], AED dose reduction or discontinuation [40, 41], psychotropic drug use (e.g., APDs or ADDs) with potential seizure-inducing effects [42, 43], premedication with xanthine derivatives other than caffeine [44], and adjusting pulse width [45].

The expert opinions on DECs in this study may not fully align with the evidence described above. Their opinions may be affected by the presence of a clear theoretical background despite limited evidence (dose reduction or discontinuation of drugs with anticonvulsant effects, such as BZRA and AEDs), concerns about adverse events (anxiety about the use of ketamine, which is not indicated for the treatment of psychiatric disorders in Japan), and simplicity in approaches (e.g., hyperventilation and anesthetic dose reduction).

Some differences were observed in the ratings of approaches that should be constantly performed and those to be performed for each disease or condition. The results of rating the approaches for patients with mood disorders were similar to those for patients with schizophrenia. However, BZRA dose reduction and discontinuation were ranked slightly lower for patients with catatonia than for patients with mood disorders or schizophrenia. This suggests that ECT experts consider BZRA a common treatment for catatonia, similar to ECT [46, 47].

Conversely, for patients at high risk of cognitive impairment, discontinuation of BZRA and switching from BL electrode placement to RUL electrode placement ranked higher, indicating that BZRA may impair cognitive function [48] and that ECT with RUL electrode placement may have a relatively less damaging effect on cognitive function than ECT with BL electrode placement [49].

For patients at high risk of cardiovascular events, premedication with xanthine derivatives and the use of psychotropic drugs with potential seizure-inducing effects were ranked lower, possibly because xanthine derivatives cause adverse events like palpitations and tachycardia [50, 51], and some APDs known to reduce the seizure threshold are associated with cardiovascular risks [52–54].

Overall, this study highlights unique aspects of ECR clinical practice in Japan, influenced by both clinical evidence and the practical realities of expert opinion.

First, for the approaches associated with anesthesia, hyperventilation was ranked very high overall because of its simplicity and safety for anesthesiologists, whereas switching anesthetics was ranked low. However, clinicians should be aware that hyperventilation increases the risk of coronary and cerebral vasoconstriction [55–57]. In addition, the lack of numerical indicators to guide the degree of hyperventilation is another issue in clinical practice. Meta-analyses have evaluated switching anesthetics from propofol to barbiturates, the combined use of remifentanil, and switching anesthetics to ketamine. The results showed that these approaches are all considered second-line strategies. This may reflect the fact that in Japan, psychiatrists have limited input on the selection of anesthetics, as anesthetic selection is often left to anesthesiologists at many facilities that provide ECT in Japan. Further investigations are required to evaluate these approaches.

Second, concerns regarding electrode placement and parameters include the infrequent use of RUL electrode placement and frequent adjustments to pulse width. The use of RUL electrode placement is not ranked high except when it is used for patients at high risk of cognitive impairment. This may be attributed to the fact that it is less commonly used in Japan than in Europe and the United States [13]. We hope that the various electrode placements will be adopted in Japan in the future. However, adjusting the pulse width is considered a second-line strategy across all conditions despite the limited evidence. In particular, many reports have been published on the efficacy of lengthening the pulse width in DECs in Japan [13, 45, 58, 59]. This may be influenced by the default pulse width of 0.5 ms on the Thymatron® System IV (Somatics), which is the only available device in Japan, shorter than the standard 1.0 ms in other countries.

Finally, there are concerns regarding the treatment approaches for patients with catatonia. The use of flumazenil is not considered a first-line or subsequent strategy under any condition. As the level of evidence for the use of flumazenil was low, expert consensus on the position of its use in this study was generally reasonable. However, no consensus was reached on its use, specifically in DECs with catatonia. In treating catatonia, BZRA use is common [46, 47]. Since the abrupt discontinuation of BZRA before ECT causes withdrawal symptoms [60], BZRA is often used concomitantly at certain doses during the administration of ECT. Although the use of flumazenil might be considered in such cases, this was not reflected in the expert consensus reached in our study. Further investigation is needed to evaluate approaches for patients with catatonia treated with BZRA.

This study has several limitations. First, guidelines were developed based on expert consensus on ECT, which generally offers a low level of evidence. Many of the clinical questions included in this study have not yet been scientifically addressed, highlighting the need for further investigation. Second, all the experts who participated in this study were Japanese physicians who had undergone similar training, which may limit the generalizability of the results. Third, the committee arbitrarily selected these approaches. Fourth, this survey was conducted at facilities in Japan, and it primarily reflects the unique circumstances of the ECT environment in Japan. For example, in Japan, the half-age dosing strategy is still more common than the titration method. Additionally, stimulation doses are limited to a maximum of 504 mC. The survey responses were obtained under these circumstances, so they may not be generalizable to other countries or regions. Fifth, the scenario in this questionnaire did not specify whether the case involved acute ECT or maintenance/continuation ECT. It is assumed that most respondents answered based on acute ECT, as many DECs are likely to occur when the threshold rises due to frequent ECT sessions. However, it cannot be ruled out that some respondents answered assuming a case transitioning from acute ECT to maintenance/continuation ECT. Sixth, a decrease in ECT session frequency was not included as an approach in this questionnaire. Reducing session frequency has been reported to be effective in lowering seizure thresholds [61]. However, it was not adopted as an approach because it is not well known in Japan. Further investigation on this point is necessary in the future. Finally, the categorization of responses (i.e., 1–3 points [disagree], 4–6 points [neutral], and 7–9 points [agree]) and the analytical methods were somewhat subjective.

## Conclusions

This study revealed that ECT experts in Japan select approaches for DECs based on patient diseases, clinical characteristics, theoretical background, evidence, presence of risks, simplicity of the technique, and circumstances surrounding the ECT. Despite the limited evidence, the recommendations provided by this study may be useful in clinical decision-making in regions where only ECT devices with a maximum stimulus charge of 504 mC are approved. In addition, in areas where devices with a maximum stimulus charge of over 504 mC have been approved, these recommendations may contribute to the reduction or avoidance of adverse events associated with the use of high charges. However, the determination of the DECs should be made carefully, considering not only the reduction in seizure duration but also whether a seizure is occurring and the overall quality of the seizure. These recommendations should be further evaluated through RCTs using different approaches (e.g., on flumazenil use in catatonia). In addition, a cross-national study is needed to validate the consensus among Japanese experts globally. Therefore, further research in this area is required.

## Supplementary Information

Additional file 1: Title of data: Questionnaire and responses. Description of data: Details of the questionnaire and questions.

Additional file 2: Title of data: Details of approaches. Description of data: List of approaches that have been reported to be effective for DEC.

Additional file 3: Title of data: Approaches for patients with mood disorders undergoing difficult-to-induce-seizures electroconvulsive therapy. Description of data: Results of the answers to Q2.

Additional file 4: Title of data: Approaches for patients with schizophrenia undergoing difficult-to-induce-seizures electroconvulsive therapy. Description of data: Results of the answers to Q3.

Additional file 5: Title of data: Approaches for patients with catatonia receiving difficult-to-induce-seizures electroconvulsive therapy. Description of data: Results of the answers to Q4.

Additional file 6: Title of data: Approaches for patients at high risk of cognitive impairment undergoing difficult-to-induce-seizures electroconvulsive therapy. Description of data: Results of the answers to Q5.

Additional file 7: Title of data: Approaches for patients at high risk of cardiovascular events undergoing difficult-to-induce-seizures electroconvulsive therapy. Description of data: Results of the answers to Q6.

## Data Availability

The data that support the findings of this study are openly available in Figshare at https://figshare.com/articles/dataset/ECT-EC_questionnaire/27124545.

## Abbreviations

ADD: Antidepressant drugs
AED: Antiepileptic drugs
APD: Antipsychotic drugs
BL: Bilateral
BZRA: Benzodiazepine receptor agonist
CI: Confidence interval
DEC: Difficult-to-induce-seizures electroconvulsive therapy
ECT: Electroconvulsive therapy
RUL: Right unilateral
RCT: Randomized controlled trial
