# Supplementary material for: Approaches for difficult-to-induce-seizures electroconvulsive therapy cases (DEC): a Japanese expert consensus
Source: Ann Gen Psychiatry. 2025 Jan 12;24:2. doi: 10.1186/s12991-024-00543-9 (PMC11727425; doi:10.1186/s12991-024-00543-9)
Supplement: Supplementary file 1 — Additional file 1: Title of data: Questionnaire and responses. Description of data: Details of the questionnaire and questions. [file 12991_2024_543_MOESM1_ESM.docx]

**Additional File 1.** Questionnaire and responses

This questionnaire is designed based on the scenario that electroconvulsive therapy (ECT) is typically administered with bilateral electrode placement under propofol anesthesia at a pulse width of 0.5 ms and the half-age dosing strategy, using the Thymatron® device (maximum stimulus charge: 504 mC), a pulse-wave therapeutic device approved in Japan.

Please, imagine a situation where all the following approaches are applicable, and answer the following questions by selecting a number between 1 and 9:

1 2 3 4 5 6 7 8 9

←Disagree　 Agree→

Q1) Which approach do you think should be constantly performed to avoid situations that make seizure induction difficult?

Q2) To what extent do you agree with the selection of the following approaches for patients with general (note 1) mood disorders in difficult-to-induce-seizures electroconvulsive therapy cases (DEC)?

Q3) To what extent do you agree with the selection of the following approaches for patients with general (note 1) schizophrenia in DEC?

Q4) To what extent do you agree with the selection of the following approaches for patients with general (note 1) catatonia in DEC?

Q5) To what extent do you agree with the selection of the following approaches for patients with a high risk of cognitive impairment (note 2) in DEC?

Q6) To what extent do you agree with the selection of the following approaches for patients with a high risk of cardiovascular events (note 3) in DEC?

Note 1: The term “general” indicates that patients have no risks interfering with ECT regarding physical and cognitive functions.

Note 2: A “high risk of cognitive impairment” means that patients currently exhibit or have previously exhibited cognitive impairment including delirium.

Note 3: A "high risk of cardiovascular events" refers to cases where hemodynamic changes should be avoided, or where patients have heart disease or cerebrovascular disorders.
