# Supplementary material for: Approaches for difficult-to-induce-seizures electroconvulsive therapy cases (DEC): a Japanese expert consensus
Source: Ann Gen Psychiatry. 2025 Jan 12;24:2. doi: 10.1186/s12991-024-00543-9 (PMC11727425; doi:10.1186/s12991-024-00543-9)
Supplement: Supplementary file 2 — Additional file 2: Title of data: Details of approaches. Description of data: List of approaches that have been reported to be effective for DEC. [file 12991_2024_543_MOESM2_ESM.docx]

**Additional File 2. (Q1) Details of approaches**

(1) Discontinuation of benzodiazepine receptor agonist (BZRA)

(2) Dose reduction of BZRA

(3) Use of flumazenil

(4) Premedication with xanthine derivatives (e.g., theophylline) or caffeine

(5) Dose reduction or discontinuation of antiepileptic drugs (AEDs) or other drugs that may make seizure induction difficult

(6) Switching to or combination of psychotropic drugs (e.g., antipsychotic drugs [APDs] or antidepressant drugs [ADDs]) with potential seizure-inducing effects

(7) Dose reduction of anesthetics

(8) Stimulation timing adjustment

(9) Switching anesthetics from propofol to barbiturates

(10) Combinations with remifentanil

(11) Switching anesthetics to ketamine (alone or in combination)

(12) Ensuring hyperventilation

(13) Adjusting the pulse width

(14) Switching from bilateral (BL) electrode placement (temporal/frontal lobes) to right unilateral (RUL) electrode placement
