# Supplementary material for: Approaches for difficult-to-induce-seizures electroconvulsive therapy cases (DEC): a Japanese expert consensus
Source: Ann Gen Psychiatry. 2025 Jan 12;24:2. doi: 10.1186/s12991-024-00543-9 (PMC11727425; doi:10.1186/s12991-024-00543-9)
Supplement: Supplementary file 3 — Additional file 3: Title of data: Approaches for patients with mood disorders undergoing difficult-to-induce-seizures electroconvulsive therapy. Description of data: Results of the answers to Q2. [file 12991_2024_543_MOESM3_ESM.docx]

**Additional File 3. (Q2) Approaches for patients with mood disorders undergoing difficult-to-induce seizure electroconvulsive therapy**


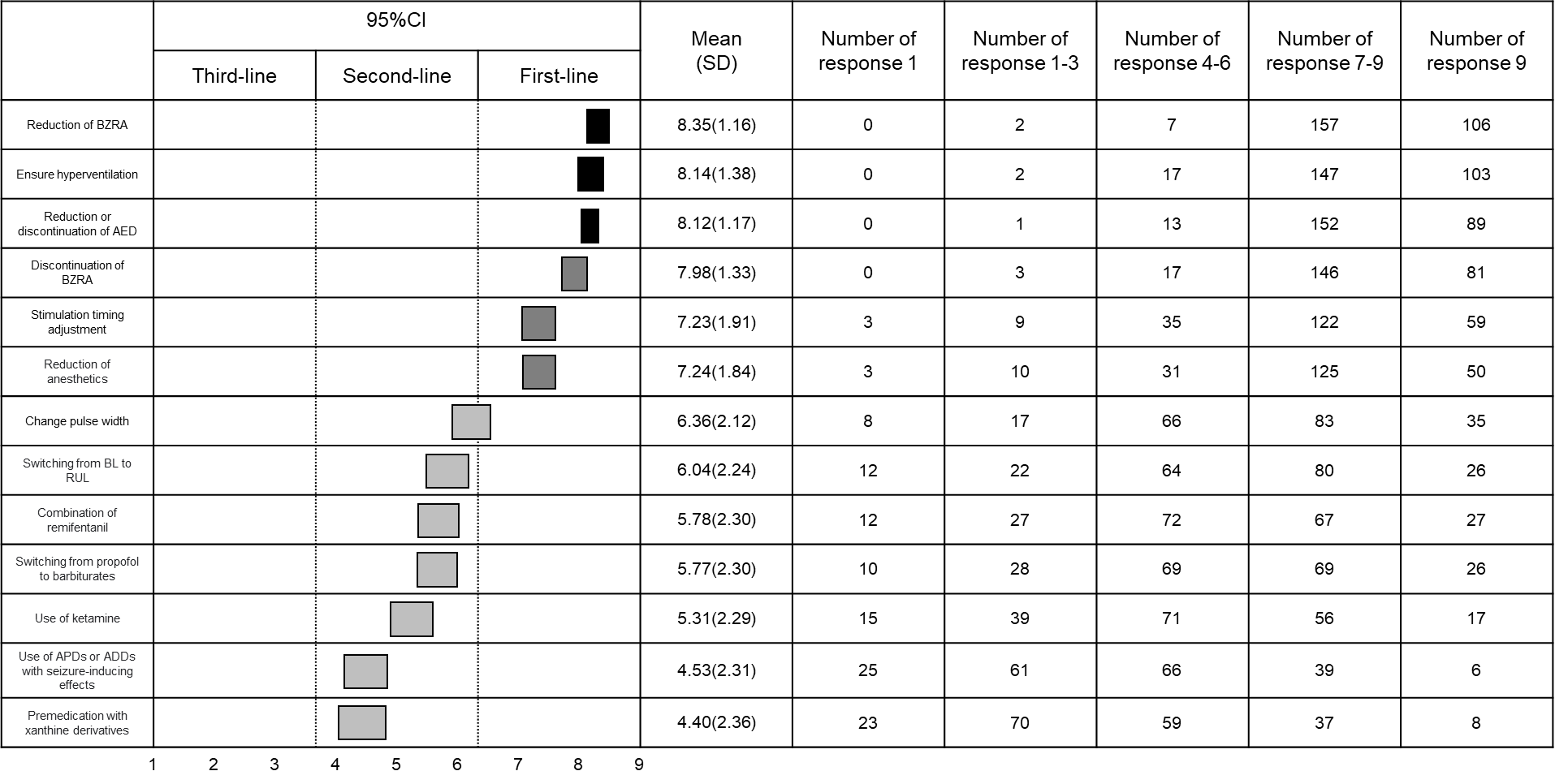


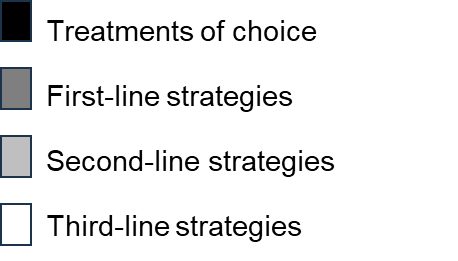


Abbreviations: CI, confidence interval; SD, standard deviation; BZRA, benzodiazepine receptor agonist; AED, antiepileptic drug; BL, bilateral; RUL, right unilateral; APDs, antipsychotic drugs; ADDs, antidepressant drugs.
